# Cryo‐EM structure of the full‐length Lon protease from *Thermus thermophilus*

**DOI:** 10.1002/1873-3468.14199

**Published:** 2021-10-18

**Authors:** Francesca Coscia, Jan Löwe

**Affiliations:** ^1^ MRC Laboratory of Molecular Biology Cambridge Biomedical Campus Cambridge UK

**Keywords:** AAA+, cell cycle, coiled‐coil, Lon, protease, unfolding

## Abstract

In bacteria, Lon is a large hexameric ATP‐dependent protease that targets misfolded and also folded substrates, some of which are involved in cell division and survival of cellular stress. The N‐terminal domain of Lon facilitates substrate recognition, but how the domains confer such activity has remained unclear. Here, we report the full‐length structure of Lon protease from *Thermus thermophilus* at 3.9 Å resolution in a substrate‐engaged state. The six N‐terminal domains are arranged in three pairs, stabilized by coiled‐coil segments and forming an additional channel for substrate sensing and entry into the AAA+ ring. Sequence conservation analysis and proteolysis assays confirm that this architecture is required for the degradation of both folded and unfolded substrates in bacteria.

## Graphical abstract

In bacteria, the hexameric ATP‐dependent size‐exclusion protease Lon performs protein quality control and also determines the concentration of key regulatory proteins via their recognition (via the N‐terminal domains), unfolding (AAA+ domains) and degradation (protease domains). The six N‐terminal domains arrange in three pairs, which produce a “collar” pore for substrate translocation towards the central channel formed by the AAA+ domains. The collar is essential for the degradation of both folded and unfolded protein substrates.

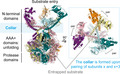

## Abbreviations

**AAA+**, ATPases Associated with diverse cellular Activities

**AMP‐PNP**, adenylyl‐imidodiphosphate

**Cryo‐EM**, electron cryo‐microscopy

**EcLon**, Lon protease from Escherichia coli

**FSC**, Fourier shell correlation

**hLon**, Lon protease from Homo sapiens

**NTD**, N‐terminal domain

**TRIS**, Tris(Hydroxymethyl)aminomethane

**TtLon**, Lon protease from *Thermus thermophilus*

**YpLon**, Lon protease from *Yersinia pestis*

Lon is a ubiquitous ATP‐dependent protease that provides housekeeping functions via the degradation of misfolded proteins and also coordinates key processes through targeted proteolysis of folded proteins [1]. In *Escherichia coli,* and most Gram‐negative bacteria, Lon controls cell division in response to DNA damage by targeting SulA, an inhibitor of FtsZ polymerization [2]. In *Caulobacter crescentus,* Lon governs the cell cycle by modulating the concentration of the DNA replication initiation factor DnaA [3, 4, 5]. Moreover, Lon is involved in the regulation of bacterial metabolism and toxin–antitoxin systems, and it is critical in many cells for survival and virulence under stress conditions [6, 7, 8]. It follows that bacteria in which *lon* has been deleted show cell division phenotypes and also a high sensitivity to antibiotics and UV radiation, making Lon a potential target for the development of antimicrobial agents [9, 10]. Eukaryotic Lon protease is of bacterial origin, it is located in mitochondria and plays a crucial role in ageing, transcription and other fundamental organelle functions in health and disease [11]. Archaeal Lon is the only family member bound to the membrane and it is important for cell viability [12, 13].

Lon protease is composed of three major domains: (a) the N‐terminal domain (NTD) recognizing substrates, (b) the hexameric AAA+ (A) domain with unfolding activity and iii) the C‐terminal serine protease (P) domain, which hydrolyses substrates [14]. Previously determined structures of bacterial and eukaryotic hexameric A‐P Lon truncations without the NTD show that, in the absence of substrate, Lon's hexameric ring adopts an open conformation, while upon substrate engagement, it changes into a closed but asymmetric ring conformation [15, 16] In the closed, substrate‐engaged form, sequential ATP hydrolysis in contiguous subunits around the ring drives the progressive translocation of unfolded substrates from the AAA+ channel towards the protease domains via the binding to a ‘staircase’ of aromatic residues. This mechanism is likely to be conserved in all Lon proteases and is related to the rotary treadmilling mechanism of other AAA+ translocases [17]. The N‐terminal domains show much larger sequence divergences across species and kingdoms than the A and P domains, and this is possibly related to organism‐specific substrate recognition requirements. The crystal structure of the *E. coli* NTD [18] shows a globular domain followed by a long α‐helical region, the ‘connecting helix’ (comprising approximately 50 residues), while no atomic structures of eukaryotic Lon NTDs are available. Although the importance of the NTDs has been characterized by biochemical studies in relation to a set of specific substrates (such as HspQ, DnaA and SulA), its arrangement and role within the enzyme's overall structure and reaction cycle have remained unclear [3, 19, 20].

Here, we have determined the complete three‐domain structure of *Thermus thermophilus* Lon at an overall resolution of 3.9 Å by cryo‐EM in a closed hexameric, substrate‐engaged state. While clearly being less well ordered, the cryo‐EM data enabled us to model the position of all six N‐terminal domains. The NTDs form a pseudo threefold ensemble on top of the AAA+ ring and control access to the AAA+ translocation channel. In addition, we performed degradation assays of known unfolded and folded substrates of *E. coli* Lon, verifying the importance of the NTD.

## Materials and methods

### Protein expression and purification

Synthetic genes for full‐length Lon protease from *Thermus thermophilus* (Uniprot Q72KS4, TtLon) and *Escherichia coli* (Uniprot P0A9M0, EcLon) were subcloned into the bacterial expression vector pOPINS‐UBE3C, bearing an N‐terminal 6X‐histidine tag followed by SUMO [21]. The plasmids were transformed into chemically competent C41(DE3) *E. coli* cells and 4L culture were grown in 2xYT medium at 37 °C in the presence of 50 µg·mL^−1^ kanamycin. Protein expression was induced at OD_600_ of 0.6 with 1 mm IPTG, and cells were grown overnight at 16 °C. Cells were harvested by centrifugation at 5000 × ***g***, re‐suspended in 100 mL buffer A: 50 mm Tris/HCl, 200 mm NaCl, pH 8.0, supplemented with lysozyme, DNAse, RNase (Sigma‐Aldrich/Merck Millipore, Burlington, MA, USA) and lysed with a cell disruptor (Constant Systems). The soluble fraction was isolated by centrifugation at 100 000× ***g*** for 1 h and loaded onto a gravity column prepacked with 2 mL of Ni‐NTA beads (Qiagen, Hilden, Germany) pre‐equilibrated with buffer A. After extensive washes with buffer A, the beads were incubated with the recombinant GST‐tagged SENP protease [21], at 4 °C for 2 h, to cleave the SUMO tag. Subsequently, untagged and unmodified Lon protease was recovered in the flow‐through via several column washes with buffer A. Complete tag removal was verified by SDS/PAGE. Untagged Lon protease was concentrated by ultrafiltration to 5 mg·mL^−1^ and further purified by size‐exclusion chromatography using a Superose 6 Increase 10/300 GL column (Cytiva, Marlborough, MA, USA), equilibrated in buffer A, resulting in a pure homo‐hexameric complex, according to a column calibration and as verified by negative staining electron microscopy. Since EcLon formed a mixture of hexamers and dodecamers on EM grids, cryo‐EM analysis was pursued using TtLon, while activity assays were performed using EcLon. The N‐terminally truncated mutant EcLon^253‐784^ was produced as described for full‐length EcLon and resulted in a purely hexameric assembly, without dodecamers, as previously reported for other similar mutants [16].

### Cryo‐EM grid preparation

3 µL of Lon at a concentration of 0.8 mg·mL^−1^, in buffer A supplemented with 1 mm AMP‐PNP and 5 mm MgCl_2_ were applied to freshly glow‐discharged (40 mA, 1 min) Quantifoil Cu/Rh 200 mesh R2/2 grids. The same Lon sample, diluted 10 times in buffer A (Lon concentration 0.1 mg·mL^−1^), was applied to graphene oxide (GO) grids, prepared as previously reported [22] using as support Quantifoil Cu/Rh 200 mesh R2/2 grids. The grids were blotted and plunge‐frozen in liquid ethane using a Vitrobot Mark IV (Thermo Fisher Scientific, Waltham, MA, USA). For both grid types, images were acquired on a K2 Summit detector (Gatan) in counting mode mounted on a Titan Krios G3 (Thermo Fisher Scientific) electron microscope at 300 kV. A Quantum GIF energy filter (Gatan) was used with a slit width of 20 eV to remove inelastically scattered electrons. Forty movie frames were recorded per image, using a fluency of 1.0 electron per Å^2^ per frame, for a total accumulated dose of 40 electrons per Å^2^ per image, at a pixel size of 1.1 Å on the specimen. Further details are presented in Table 1.

**Table 1:** Cryo‐EM and model data.

Statistics	
Sample	Lon from *T*. *Thermophilus*
NCBI Database Ids	Uniprot: Q72KS4
Construct	Full‐length, untagged
Method	Cryo‐EM
Data collection	
Microscope	Titan krios G3
Detector	K2 Summit
Acceleration energy	300 kV
Symmetry	C1
Data	
Resolution (Å)	3.91
Images	44449
Pixel Size (Å)	1.1
Defocus Range (µm)	0.5–4
Fluence	40 electrons/Å2
Applied B‐factor	−96.81
Model Refinement	
R_work_/R_free_	0.40/0.40
Bond length rmsd (Å)	0.007
Bond angle rmsd (°)	1.374
Ramachandran (%)	
Favoured	93.66
Outliers	0.06
MolProbity Clashscore	7.14 (86th percentile)
PDB, EMDB Ids	7P6U, EMD‐13232

### Cryo‐EM image processing

**Fig. 1:**
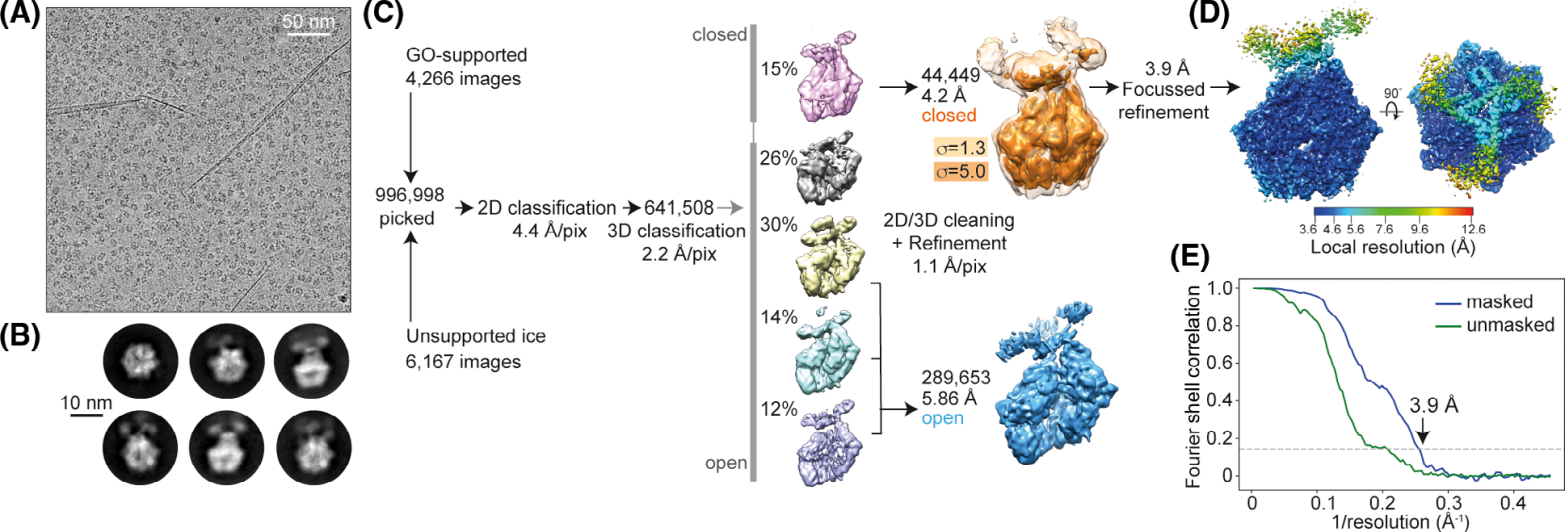
Cryo‐EM image processing of Lon from TtLon. (A) Typical micrograph of Lon from *T*. *thermophilus* (TtLon) on graphene oxide (GO) grids. The dark lines are the edges of GO flakes. (B) Representative 2D class averages. (C) Image‐processing workflow to obtain maps for atomic model building (overall 4.2 Å resolution; 3.9 Å resolution within A‐P and parts of the NTDs). (D) Local resolution of the substrate‐engaged TtLon map (E) Fourier shell correlation (FSC) of masked and unmasked maps, the dotted line indicates FSC = 0.143.

The image‐processing procedures are graphically represented in Fig. 1. Movie frames were corrected for gain using a reference, motion‐corrected and dose‐weighted using MOTIONCOR2 [23]. Aligned micrographs were used to estimate the contrast transfer function (CTF) in Gctf [24]. All subsequent image‐processing steps were performed using single‐particle reconstruction methods in RELION 3.0 [25, 26]. Particles were initially manually picked to generate 2D class references for automated picking in RELION. The whole dataset of automatically picked images was extracted with 4 × 4 binning, and two rounds of reference‐free 2D classifications were performed. In order to compensate for preferred orientation adopted by Lon on the grids, we combined two datasets collected on unsupported ice and on graphene oxide‐supported grids. The particles belonging to the best 2D classes were extracted with 2 × 2 binning and subjected to 3D classification using as initial model the structure of *Bacillus* *subtilis* Lon A‐P domains (PDB ID 3M6A) [27]. The majority of the particles presented an open‐ring conformation, similar to previously reported substrate‐free Lon structures [15, 16]. About 15% of the dataset presented a closed conformation very similar to the substrate‐engaged structure of *Yersinia* *pestis* (PDB ID 6ON2) [15], but showing additional map regions likely accounting for the six N‐terminal domains. After further 3D classification, and overall refinement at a pixel size of 1.1 Å (unbinned), we performed Bayesian polishing, per‐particle CTF and tilt correction, achieving a map at an overall resolution of 4.2 Å (Fourier shell correlation (FSC) at 0.143), much better resolved on the C‐terminal portion 250‐795. Furthermore, we performed focussed refinement with a mask to reach an overall resolution of 3.9 Å. From this map, we tried to improve the N‐terminal domain resolution by focussed refinement and signal subtraction, but this did not significantly improve the map, probably due to the small size of the N‐terminal domain and pseudo 3‐fold symmetry.

**Fig. 2:**
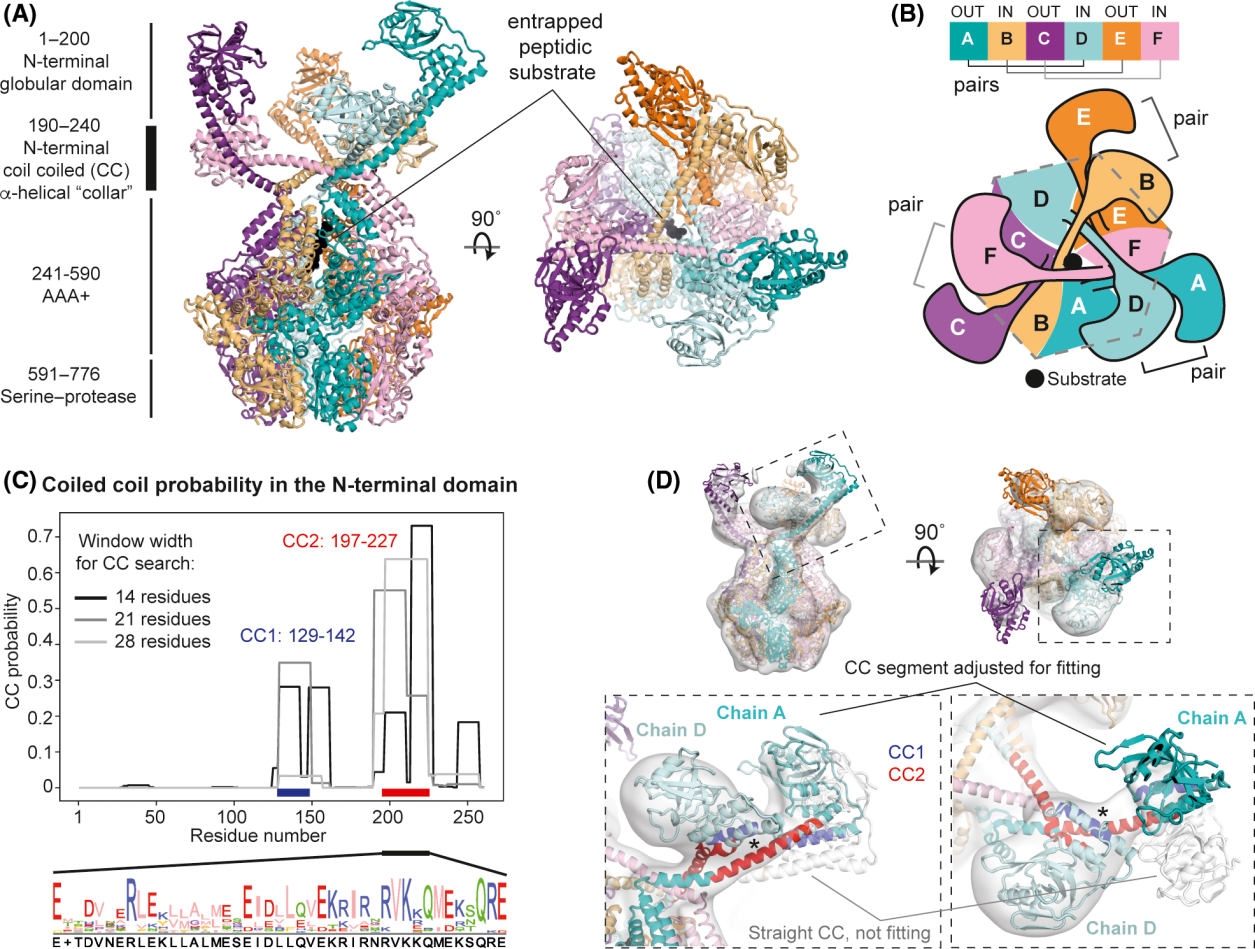
Architecture of the N‐terminal domains (NTDs) of Ttlon. (A) Cartoon representation of the closed TtLon structure. The substrate in the channel is coloured in black and shown as spheres. (B) Schematic of the arrangement of the NTDs: in consecutive chains, the connecting α‐helix (residues190‐240) points alternatingly inwards and outwards the central axis, thereby forming three pairs of NTD domains with a pseudo 3‐fold symmetry, and an additional channel above the main AAA+ channel, named here the ‘collar’. (C) Coiled‐coil probability within the NTD: the major CC regions are between residues 129‐142 (CC1) and 197‐227 (CC2). (D) Close‐up of one of the three NTD pairs composed of chains A and D, fitted into the TtLon map (at 4.2 Å, Gaussian filtered). The interaction between CC1(blue) of chain D and CC2 (red) of chain A is shown with an asterisk and drives the bending of the connecting helix composed of residues 190‐240. The same straight helix from the isolated EcLon NTD crystal structure (PDB ID 3LJC) [18] does not fit the map (white ribbon).

### Model building and refinement

Model building was conducted using two separate maps: (a) map 1, at 3.9 Å resolution, masked around a small portion of the N‐terminal domains, the AAA+ and protease domains; (b) map 2, at 4.2 Å, obtained with a larger mask enclosing all of the N‐terminal domains, which was blurred applying a 1.1 Å Gaussian filter in chimera [28]. A homology model of the TtLon A‐P domains (residues 247‐775) was generated from PDB ID 6ON2 [15] in SWISS MODEL [29], followed by a rigid body fit in Chimera [28] in map 1 (map to model correlation 0.8). A peptide substrate of unknown sequence (indicated as polyalanine, chain S) was identified in the central AAA+ pore. The bound nucleotide AMP‐PNP was clearly visible and replaced ATP and ADP moieties present in the starting reference structure of YpLon [15]. To interpret the N‐terminal region (residues 1‐246), a rigid body fit in map 1 was performed with a homology model generated with SWISS MODEL, starting from the structure of the *E. coli* N‐terminal domain (PDB ID 3LJC) [18]. While the last helical portion (residues 229‐240) fitted map 1 well, the rest of the helix and the globular domain (residues 1‐189) did not fit map 2. From the map and the coiled‐coil probability calculated in COILS [30], we surmised a superhelical twist of the ‘connecting’ α‐helix 190‐240 of the NTDs. We thus calculated a theoretical coiled‐coil structure for this portion using CCbuilder 2.0 [31] and replaced the straight α‐helix present in the initial model. The new model containing a coiled‐coil α‐helix describes map 2 well. Since the side chains of the region 1‐247 were not well defined due to resolution limitations, we set occupancy to zero for this region to indicate a degree of uncertainty. The overall model of TtLon was refined in COOT [32] and Phenix [33] (phenix.real_space_refine) and deposited in the Protein Data Bank (PDB ID 7P6U, EMD‐13232).

### Sequence conservation analysis

**Fig. 3:**
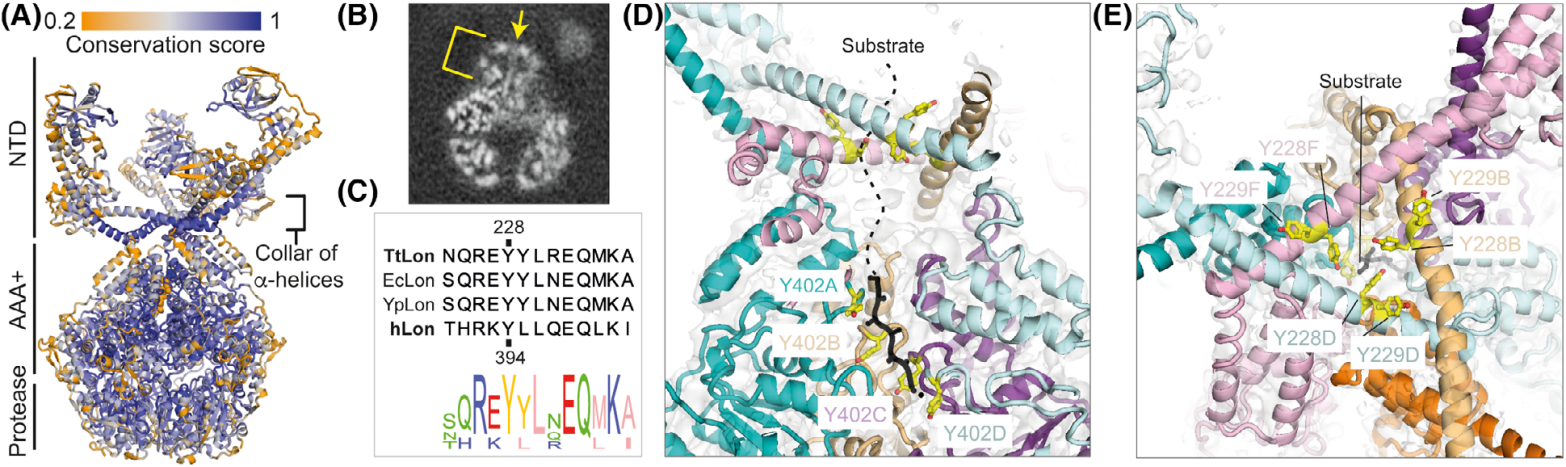
The role of the Lon N‐terminal domain (NTD) arrangement in substrate processing. (A) Cartoon of TtLon, colour‐coded according to residue conservation. The collar region formed by the intertwined connecting a‐helices of the six NTDs is highly conserved, as are the AAA+ and protease domains. (B) Longitudinal section of the experimental cryo‐EM map showing a map area that we presume is a substrate crossing the NTD collar before reaching the AAA+ ring. (C) Sequence alignment of bacterial TtLon, EcLon, YpLon to hLon; Y228 in TtLon corresponds to Y394 in hLon. (D) Substrate entry via the NTD into the AAA+ channel of TtLon. The ‘tyrosine doublets’ (Y228‐Y229) from chains B, D and F, forming the central pore of the collar, may facilitate or control substrate entry and translocation into the AAA+ channel, where Y402 from consecutive chains A to D pull the substrate towards the protease domain for hydrolysis. (E) Top view, 90° rotated from panel D.

A set of 100 sequences for bacterial Lon proteases was aligned with BLAST [34]. We discarded redundant sequences and performed further alignment with MSA‐probs [35]. Per‐residue conservation scores were calculated according to Capra *et al*. [36] and a custom Python script was used to visualize them in pymol 2.3.0 (Fig. 3A).

### Degradation assays

Peptide degradation by EcLon^FL^ and EcLon^253‐784^ was assayed by SDS/PAGE after incubation at 37 °C for 1 h. Reactions contained: Lon (hexamer 200 nm), 10 µM of protein substrate, an ATP regeneration system (16 mm creatine phosphate, 0.32 mg·mL^−1^ creatine phosphokinase [both Sigma‐Aldrich]), in a final buffer composed of 50 mm Tris/HCl pH8.0, 200 mm NaCl, 10 mm MgCl_2_, 5 mm ATP (Jena Bioscience), 2 mm DTT. Casein was purchased from Sigma‐Aldrich, while HspQ from *E. coli* (Uniprot P0AB20), PinA (Uniprot P07068) and FolA‐Sul20 were produced recombinantly, as described previously [37, 38, 39].

## Results

### Architecture of *Thermus thermophilus* Lon protease

In order to produce untagged Lon proteases, we fused a set of bacterial Lon genes with an N‐terminal SUMO tag for bacterial overexpression, to obtain completely unmodified proteins after cleavage with SUMO protease. Amongst the screened recombinant bacterial Lon proteases, the protein from *T*. *thermophilus* (TtLon) provided the most stable and homogenous hexameric assembly. We therefore used TtLon for cryo‐EM studies in the presence of the slowly hydrolysable ATP analogue AMP‐PNP. An overview of the cryo‐EM data and image‐processing workflow is shown in Fig. 1. We collected two datasets, one on unsupported holey grids and one on graphene oxide (GO)‐supported grids (Fig. 1A) that, when combined, produced a broad orientation distribution of the resulting particles, as judged by the appearance of 2D class averages (Fig. 1B). The 2D averages show a clear hexameric density decorated with an additional moiety, which we presumed was formed by the NTDs. It was not possible to determine whether Lon was in an open or closed ring conformation based on the 2D averages. We then used as starting model the open A‐P ring crystal structure of *Bacillus* *subtilis*(PDB ID 3M6A) [27] and could clearly identify both open and closed conformations in the dataset by 3D classification (Fig. 1C). However, only the subset corresponding to the closed conformation resulted in a well‐resolved map at 4.2 Å resolution. This map showed six extra map areas accounting for the NTDs' globular domains at low map contour values (σ = 1.3). In contrast, the particles in the open ring conformation were probably a mixture of many different states, due to the flexibility of the less compact A‐P ring. To improve the resolution of the closed‐conformation TtLon map, we performed focussed refinements centred on the A‐P ring and also the most resolved region of the NTDs, finally reaching an overall resolution of 3.9 Å, with highest resolutions observed within the AAA+ domains (Fig. 1D–E). Both TtLon maps from the focussed refinements were used for model building and refinement, as detailed in the Methods section and Table 1.

The A‐P ring of TtLon closely resembles the *Yersinia* *pestis* Lon (YpLon) structure (PDB ID 6ON2), obtained previously in the presence of ATP and a folded substrate (RMSD = 2.6 Å). In our map, (un‐hydrolysed) AMP‐PNP could be identified in all ATPase pockets, except in chain F, which corresponds to the site where ATP is completely hydrolysed to ADP in YpLon (Fig. S1A). In addition, we could identify an elongated map density that is most likely a peptide or mix of peptides, entrapped in a similar position to the suspected substrate in the YpLon structure at the entrance of the AAA+ central pore (Fig. 2A–B). The substrate‐engaged hLon protease (PDB ID 7KSM) [40] is less similar, but both ATPase pockets and suspected substrate binding are also highly conserved (hLon RMSD with TtLon = 4 Å), as shown in Fig. S1B–E.

While the folded portion of the substrate and the NTDs were not visible in the YpLon map, in our TtLon maps we were able to locate all six NTDs, which show a pseudo threefold arrangement. A similarly shaped NTD portion had previously been observed at low‐resolution in human Lon (hLon) by Kereïche *et al*. [41]. In TtLon's consecutive chains around the ring, the long α‐helices connecting the NTDsto the A domains (residues 190‐240) point alternatingly inwards and outwards with respect to the central AAA+ ring axis. This leads to i)the globular domains of the NTDs from chain x and chain x+3coming close, forming three chain pairs: A‐D, B‐E, C‐F (Fig. 2B) and ii) the formation of a central open α‐helical ‘collar’ above the central axis. This arrangement is stabilized by interactions between the two predicted coiled‐coil regions in each NTD: 129‐142 (CC1) and 197‐227 (CC2). CC2 from chains A, C and E interact with CC1 from chains D, F and B, respectively, thereby inducing a kink in the NTD's connecting α‐helix (Fig. 2C). While a rigid‐body fit of the *E. coli* NTD crystal structure (PDB ID 3LJC), containing a straight connecting α‐helix positions the globular domains outside our map, modelling the connecting α‐helix as a coiled‐coil instead results in a good fit (Fig. 2D and Fig. S2). Apart from major interactions between the connecting helices in chains x and x + 3, near the central pore, these two helices are sandwiched by a short portion of the connecting helix in chain x + 5 (Fig. S2). In the collar region, the positions of the amino acid residues in the model are indicative, only, due to low local resolution, and consequently, the occupancies of those residues have been set to zero. According to the NTD TtLon model, the residue E240 (corresponding to *E. coli* Lon E240) is located at the interface between the NTDs' connecting helices (Fig. S2C). It has been shown previously that E240K is defective in proteolysis and we suggest that the mutant likely destabilizes the collar structure. This could also explain the formation of more dodecameric Lon by the same mutant [41, 42, 43].

### The role of the N‐terminal domain in substrate processing

The coiled‐coil α‐helical collar portion of the NTDs, assembled from the NTDs' connecting helices, is highly conserved in bacterial, as well as in eukaryotic Lon protease sequences (Fig. 3A), suggesting that this region adopts a similar conformation to the one determined here and also suggesting a general role for the collar in Lon substrate processing.

Inspecting our TtLon maps at low contour values, we noticed that a continuous substrate density departs down from the NTD collar pore (with the NTDs at the top), reaching the AAA+ channel (Fig. 3B). We surmise that the ‘tyrosine doublets’ Y228/Y229 from chains B, D and F, forming the central pore of the collar, may facilitate substrate entry and possibly also help with translocation into the AAA+ channel. The Y228/Y229 tyrosine doublet is highly conserved in bacteria. The first Y228 corresponds to Y394 in human Lon, which, when mutated to alanine, severely alters Lon activity [44] (Fig. 3C). In the TtLon AAA+ channel, the staircase of the Y402 residues from the consecutive chains A‐D presumably pulls the substrate towards the protease domains for hydrolysis, as suggested for example by the YpLon structure [15], and in agreement with other AAA+ translocases [15, 40, 45, 46] (Fig. 3D,E and Fig. S1B‐E). The structural features observed here suggest a direct coupling between the NTD collar ‘sensing and holding’ the substrate, with its progressive unfolding and translocation driven by ATP hydrolysis in the A domains. Indeed, previous studies showed that removal of the α‐helical region 232‐252 in *E. coli* Lon compromises ATPase activity and ATP‐dependent peptide translocation abilities [47].

### Protein degradation by EcLon

**Fig. 4:**
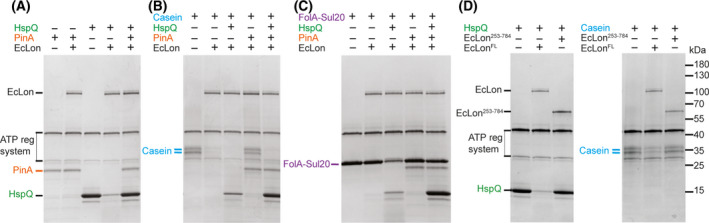
Protein degradation by EcLon is dependent on the N‐terminal domains, highlighting a role of the collar region. Proteolysis in the presence of an ATP regeneration system (reactions ran at 37 °C for 1 h) of unstructured casein and folded HspQ and FolA‐Sul20 substrates, and inhibition by the phage‐derived PinA protein were assayed by SDS‐PAGE. (A) Pin A is not degraded by Lon and inhibits degradation of HspQ. Casein (B) and FolA‐Sul20 (C) degradation is accelerated by HspQ and inhibited by PinA. In the presence of both HspQ and casein, the latter is preferentially degraded. (D) The NTD, including the collar portion containing the tyrosine doublets, is required for the degradation of both folded (HspQ) and non‐folded (casein) substrates.

Our map showed that a copurified unfolded peptide or peptide mix seems to cross the collar pore to reach the AAA+ ring. Based on this, we asked whether the collar structure is necessary for the processing of both unfolded and folded proteins and how the pseudo threefold arrangement of the NTDs' globular domains is involved in the recognition of EcLon‐specific interactors. We therefore performed degradation assays in the presence of Lon substrates and the known Lon ‘activity enhancer’ HspQ, which is also itself a Lon substrate [19]. We used the widely used model of a folded Lon substrate, FolA‐Sul20 (dihydrofolate reductase, fused to the SulA C‐terminal peptide degron, as described by Gur *et al*. [37]), unstructured casein [48] and the phage‐derived Lon inhibitor PinA [38, 49] (Fig. 4).

Apart from being a Lon substrate itself, HspQ is known to accelerate the degradation of other folded and unfolded substrates [19, 39]. In our assay, HspQ accelerated degradation of non‐folded casein, and to a larger extent of folded FolA‐Sul20, thereby confirming its enhancer function (Fig. 4B‐C). However, in the absence of the NTD (EcLon truncation including residues 253 to 784, which does not include the collar), both the degradation of HspQ and casein were impaired, making it likely that binding of HspQ is mediated by the NTD and that the collar is essential for unfolded substrates to be translocated towards the protease domains (Fig. 4D). Based on these data and on the structures of Lon and HspQ, we speculate that the HspQ trimer might bind directly to the pseudo threefold NTDs, to affect ATPase and translocation by the AAA+ ring and/or to enhance substrate access, for example by modifying the collar pore. Interestingly, PinA, binding Lon directly [50], inhibits HspQ and FolA‐Sul20 degradation while inhibiting casein degradation to a somewhat lesser extent. This could suggest that PinA competes for Lon binding with HspQ through overlapping binding sites.

## Discussion

In bacteria, ATP‐dependent Lon proteases control directly the intracellular concentrations of many important proteins, for example modulating cell division and survival in response to metabolic, chemical, thermal and DNA damage stress [51]. A range of substrates and their interactions with Lon have been studied; however, many of the substrates are also degraded by other protease systems, for example Clp [52, 53], significantly complicating analyses. A key challenge is thus to identify Lon‐specific substrates and their mechanism of recognition. The full‐length bacterial Lon structure presented here provides insights into the unique mechanism of substrate processing involving the enigmatic N‐terminal domains. In Lon, unlike in most other ATP‐dependent size‐exclusion protease systems, the NTD is part of the same polypeptide chain as the AAA+ and protease domains. As shown here, the NTD arrangement in the Lon hexamer consists of two structural features: a collar formed by the connecting helices in a coiled‐coil‐like arrangement, and a pseudo 3‐fold triplet of globular domain pairs, presumably involved in specific substrate recognition, such as with HspQ. Previous studies showed that the NTD does not form dimers *in vitro* or in the crystals of the reported structures; therefore, this arrangement, enforced by the AAA+ hexameric assembly, only occurs within the complete Lon quaternary structure [18, 27]. We show that the collar is an integral part of the translocation machinery, threading the substrate towards the AAA+. Our and previous biochemical studies on EcLon also show that this region is essential for processing of both unfolded and folded proteins [47]. As such, the collar could provide an access gating mechanism that allows, or disallows access to the AAA+ channel, depending on whether the globular domains of the NTDs have engaged with substrates.

While the amino acid sequences of the globular domains of the NTDs are often longer and are also more diverse in eukaryotes, the collar structure is likely to be conserved, as is also highlighted by the two recently reported structures of hLon protease that resolve the positions of the NTDs [44, 54]. These studies also reported a similar architecture of the NTDs and the aromatic residues within the collar channel. In one of those studies, in line with our hypothesis, mutation of one tyrosine belonging to the NTD tyrosine doublet to alanine (Y228 in TtLon, Y394 in hLon) dramatically impairs Lon activity [44].

It remains to be established how the NTDs discriminate between potentially many different Lon‐specific protein targets [55]. It seems unlikely to us that a small globular domain such as the one present in the NTD of Lon could recognize and discriminate between a large number of different substrates. A different, or perhaps indirect targeting strategy, or using more general motifs or degrons, seem more likely. Along those lines, HspQ enhances Lon activity for many substrates. Interestingly, the HspQ trimer presents a surface complementarity with the solvent‐exposed part of the collar and NTD (top). A possible explanation of HspQ’s enhancing function could be that it docks onto the NTD and ‘pushes apart’ the collar and the connecting helixes causing a constant or wider opening of the central channel, which possibly facilitates the entry of certain substrates (and itself). For this to work, HspQ itself would have to open up a central pore to let substrates access the Lon channel. Alternatively, it is also possible that HspQ shuttles substrates to Lon, acting as an ‘adaptor’ protein. However, these hypotheses will need to be investigated since there is currently no evidence to support one or the other. Our proteolysis assays suggest that the T4 phage inhibitor protein PinA could be at least partly sharing its Lon binding region with HspQ. Starting with the complete TtLon structure we report here, it will be crucial to obtain structural information on both HspQ and PinA interactions and to obtain comprehensive substrate specificity data to unravel the secrets of the Lon protease machine further.

## Author contributions

FC and JL designed the project. FC produced the proteins, collected and analysed EM data, performed biochemical assays and data analysis. JL and FC built the atomic model and wrote the manuscript.

## Supporting information


**Fig. S1**. Comparisons of ATP binding sites and substrate translocation sites in TtLon, YpLon and hLon.
**Fig. S2**. Predicted molecular interactions between the connecting helices within the NTD.

## Data Availability

The biochemical data that support the findings of this study are available in Fig. 4. The structural data that support these findings are openly available in the wwPDB at https://doi.org/10.2210/pdb7P6U/pdb (PDB ID 7P6U) and in the EMBD https://www.ebi.ac.uk/pdbe/emdb(EMD‐13232).
