# Supplementary material for: Cryo‐EM structure of the full‐length Lon protease from Thermus thermophilus
Source: FEBS Lett. 2021 Oct 18;595(21):2691–700. doi: 10.1002/1873-3468.14199 (PMC8835725; doi:10.1002/1873-3468.14199)

**Supplementary Figure 1: Comparisons of ATP binding sites and substrate translocation sites in TtLon, YpLon and hLon.** A) Left: cut-away view showing the ATPase sites, which are located between the subunits. Right: the upper panels show the TtLon EM map and the corresponding atomic nucleotide models. The lower panels show the overlap of the atomic models (colour for TtLon as in upper panels) with YpLon (blue) and hLon (dark gray) for each chain. In chain F, TtLon’s site is unoccupied while an ADP molecule is present in the other two homologue structures. B) The square indicates the region where an extended peptide substrate can be modelled in TtLon. C) Close up of the modelled substrate peptide in TtLon. D) The substrate peptide and the tyrosine residues from consecutive chains (Y402 and Y398 respectively) in TtLon (black) and YpLon (blue) superimpose very well (overall RMSD = 2.6 Å). E) In hLon, the substrate peptide is somewhat shifted with respect to TtLon, but the overall arrangement with respect to the translocating tyrosines (Y565) is conserved (overall RMSD = 4Å).

**
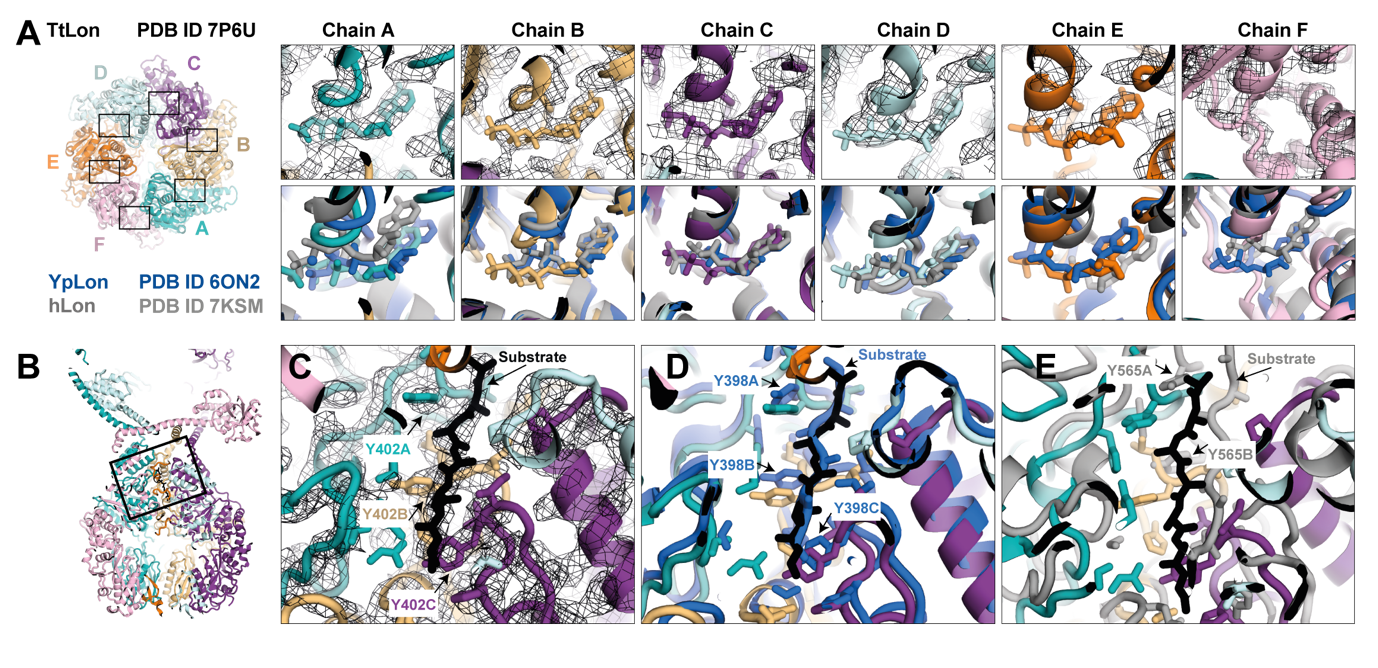
**

**Supplementary Figure 2: Predicted molecular interactions between the connecting helices within the NTD.** A) One of the three regions in the TtLon NTD where the connecting helices in chains x, x+3 (and x+5) interact (with x=1 for chain A and x=4 for chain D). Contacts involve mainly hydrophobic interactions and are located within the the predicted coiled coils stretches (see also Figure 2). B) and C) show an example of side chain interactions between subunits A, D and F. E240 is located at the interface between chain A and F and could interact with R226. Due to local resolution limitations, the NTD model is largely based on a rigid body fit and the register of the displayed side chains should be considered indicative. To indicate this uncertainty, occupancies for those residues have been set to zero.


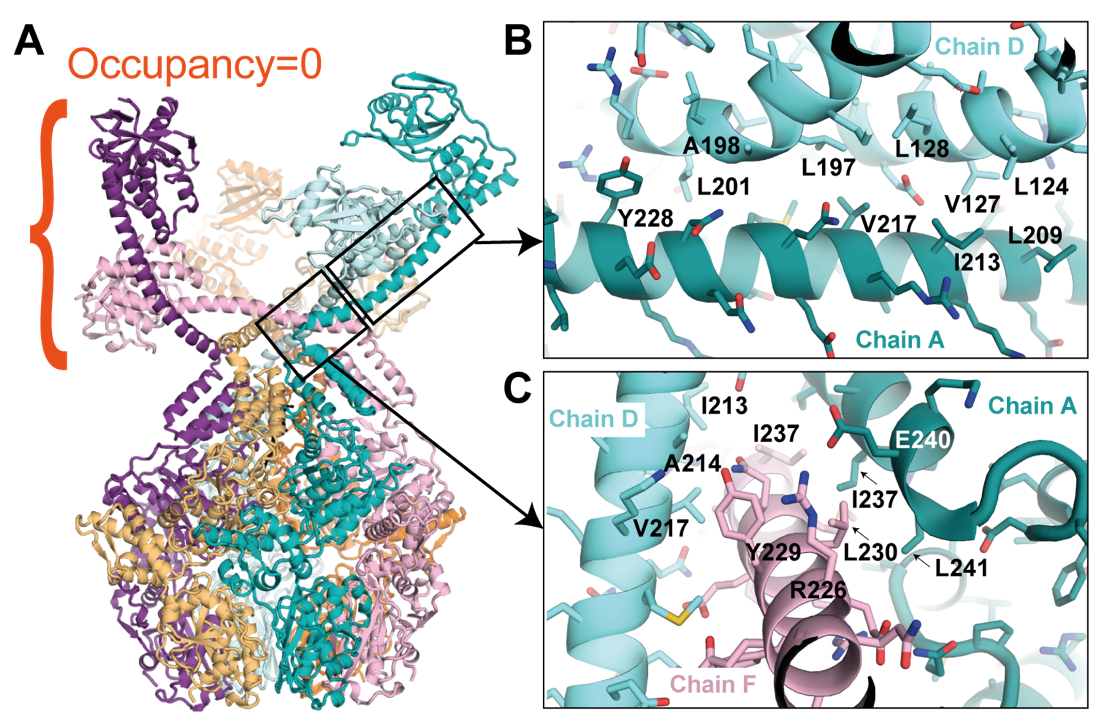

Supplement: Supplementary file 1 — Fig. S1. Comparisons of ATP binding sites and substrate translocation sites in TtLon, YpLon and hLon. Fig. S2. Predicted molecular interactions between the connecting helices within the NTD. [file FEB2-595-2691-s001.docx]
